# Bile salts enhance the susceptibility of the peach allergenic lipid transfer protein, Pru p 3, to in vitro gastrointestinal proteolysis

**DOI:** 10.1038/s41598-023-39599-0

**Published:** 2023-09-13

**Authors:** Kai Wang, Judit Gali-Moya, Maria Ruano-Zaragoza, Kathleen Cain, Giovanni D’Auria, Matthew Daly, Perdita Barran, René Crevel, E. N. Clare Mills

**Affiliations:** 1 School of Biological Sciences, Manchester Academic Health Sciences Centre, Manchester Institute of Biotechnology, 131 Princess Street, Manchester, M1 7DN UK; 2 Allergy Department, Hospital Clinic of Barcelona, 08036 Barcelona, Spain; 3 Department of Chemistry, Manchester Institute of Biotechnology, 131 Princess Street, Manchester, M1 7DN UK; 4 Department of Agricultural Sciences, University of Naples Federico II, Portici, Italy; 5 René Crevel Consulting Ltd, Suite A 82 James Carter Road, Mildenhall, IP28 7HP UK; 6 School of Biosciences and Medicine, The University of Surrey, Guildford, GU2 7XH Surrey UK

**Keywords:** Biochemistry, Immunology

## Abstract

Sensitisation to the lipid transfer protein Pru p 3 is associated with severe allergic reactions to peach, the proteins stability being thought to play a role in its allergenicity. Lipid binding increases susceptibility of Pru p 3 to digestion and so the impact of bile salts on the in vitro gastrointestinal digestibility of Pru p 3 was investigated and digestion products mapped by SDS-PAGE and mass spectrometry. Bile salts enhanced the digestibility of Pru p 3 resulting in an ensemble of around 100 peptides spanning the protein’s sequence which were linked by disulphide bonds into structures of ~ 5–6 kDa. IgE binding studies with a serum panel from peach allergic subjects showed digestion reduced, but did not abolish, the IgE reactivity of Pru p 3. These data show the importance of including bile salts in vitro digestion systems and emphasise the need to profile of digestion in a manner that allows identification of immunologically relevant disulphide-linked peptide aggregates.

## Introduction

Peach (*Prunus persica*) represents one of the major food allergens in the Mediterranean countries with about 60% of peach allergic patients being sensitised to the lipid transfer protein (LTP) allergen Pru p 3 with the prevalence of probable peach allergy ranging from ~ 1.6 to 2.0% of adults in Zurich and Madrid to almost zero in Reykjavik^1,2^. A member of prolamin superfamily, Pru p 3 is found in a range of fruits as well as in nuts, seeds, vegetables and pollen^3,4^. Previous studies have demonstrated that it is the primary sensitiser of the LTP family, although the mugwort pollen allergen, Art v 3 also contributes to primary sensitisation in some patients^5–9^.

Like other LTPs, Pru p 3 is a low molecular weight (9 kDa) α-helical lipid-binding protein involved in protection and defence of plants against microbial pathogens, possessing the conserved cysteine skeleton characteristic of the prolamin superfamily^10^. The cysteines form intra-molecular disulphide bonds in such a way as to create a hydrophobic tunnel in the centre of the protein which is capable of binding a variety of lipophilic molecules^10^. The central calyx of LTPs is known be plastic and able to accommodate molecules such as prostaglandins which possesses a cyclopentone ring^11^ with the alkyloid camptothecin recently having been proposed as the natural ligand for many LTPs including Pru p 3^12^. The protein is stable to both thermal treatment^13^ and low pH^14^ and is highly resistant to gastro-duodenal proteolysis^1,15^ retaining its IgE binding capacity after digestion^16^. Enhancing the conformational flexibility of Pru p 3 either by ligand binding^17^ or reduction and alkylation^18^ increases the protein's susceptibility to either gastroduodenal proteolysis or pepsin followed by trypsin digestion respectively. Such stability is thought to play a role in the ability of Pru p 3 to sensitise individuals and contribute to its ability to trigger severe systemic reactions^3^.

Bile salts are well-characterised biosurfactants which are produced in the liver and released into the duodenum following food ingestion, cholate and deoxycholate being two major bile salts which are conjugated to either taurine or glycine before being secreted^19^. They play a crucial role in the solubilisation and absorption of lipids and conjugated bile acids having been shown to enhance the digestion of several proteins^20^. Since fatty acid binding has previously been shown to enhance the susceptibility LTPs to digestion^17^ it was hypothesised that bile salts may also bind to Pru p 3 and enhance its susceptibility to gastroduodenal proteolysis. Therefore, the effect of bile salts on the digestion of Pru p 3 and its IgE-reactivity was investigated using an in vitro model of conditions in the duodenum (pH 6.5) and intestine (pH 8.0). This comprised gastric digestion using a pepsin test followed by an intestinal digestion test performed at either pH 6.5 or pH 8.0, to replicate conditions found in the duodenum and ileum respectively. The intestinal digestion test employed a combination of trypsin and chymotrypsin with either a high^21^ or a low protease: protein ratio to simulate conditions found in adults and infants respectively^22^.

## Results

### Enhancement of gastrointestinal digestibility of Pru p 3 by bile salts

**Figure 1:**
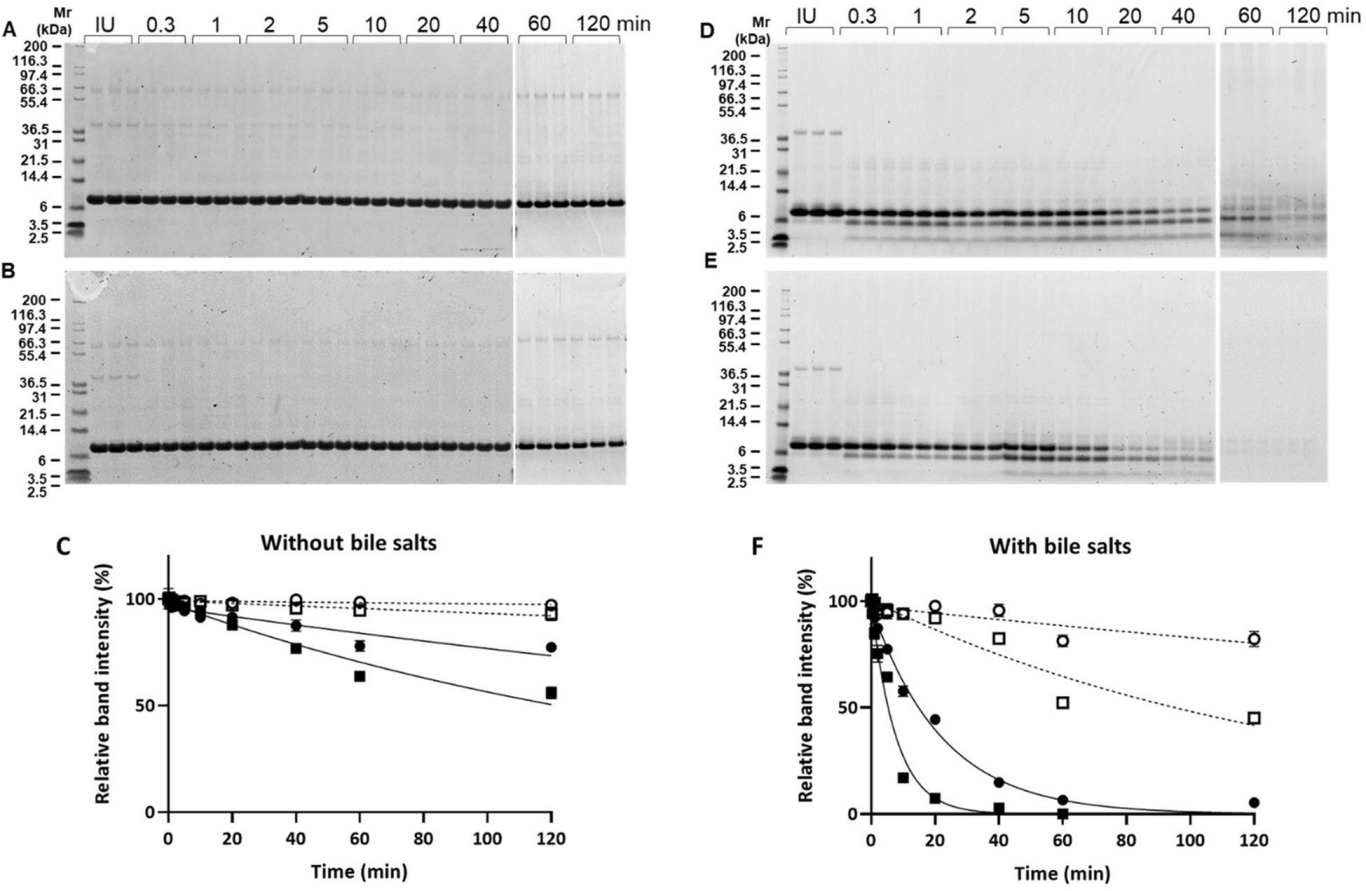
Digestion of Pru p 3 under different in vitro gastrointestinal conditions. Digestions were performed in the absence (**A**–**C**) and presence (**D**–**F**) of bile salts. Panel (**A**,**D**) high enzyme test, pH 6.5; panel (**B**,**E**) high enzyme test, pH 8.0. Exponential curves were fitted to relative band intensity obtained from densitometric analysis of SDS-PAGE gels (**C**,**D**). Low enzyme tests at either pH 6.5 (open circle) or pH 8.0 (open square) and high enzyme tests at either pH 6.5 (closed circle) or 8.0 (closed square).

Initially the impact of bile salts on the digestibility of Pru p 3 in an in vitro gastrointestinal model employing the high and low enzyme tests at either pH 6.5 or 8.0, was assessed using SDS-PAGE and HPLC (Fig. 1; Supporting information, Figs. S1, S2). Pru p 3 was resistant to gastro-intestinal proteolysis in the absence of bile salts (Fig. 1A,B; Supporting information, Fig. S1A,B), densitometric analysis showing that around 40% of the protein was digested in the high enzyme test at pH 8.0 whilst it was undigested at pH 6.5 (Fig. 1C). However, when bile salts were included in the duodenal (pH 6.5) and intestinal (pH 8.0) digestion tests Pru p 3 breakdown increased such that only a trace of the parent protein remained at either 120 min (pH 6.5) or 40 min (pH 8.0) in the high enzyme test supplemented with 4 mM bile salts (Fig. 1D,E). Digestion in the low enzyme test supplemented with 1 mM bile salts was slower and resembled that of the high enzyme test performed without bile salts (Supporting information, Fig. S1C,D).

Based on densitometric analysis the half-life of Pru p 3 was calculated for the different digestion conditions (Table 1) and found to range from 5.4 to 427.9 under the different conditions employed. Inclusion of bile salts increased the rate and extent of digestion in all the in vitro digestion conditions studies, which was reflected in a 20–25-fold decrease in half-life in the high enzyme test performed at pH 8.0 with 4 mM bile salts, addition of 1 mM bile salts reducing it in the low enzyme activity test at pH 8.0 from infinity to more than 90 min (Table 1).

**Table 1:** Rate constants and half-life of Pru p 3 and a Mr ~ 7 kDa fragment following gastrointestinal digestion performed under different conditions. *ND* not determined.

Test type	pH	Bile salts (mM)	Pru p 3		Mr ~ 7 kDa fragment
			K (min^−1^)	t_½_ (min)	K (min^−1^)
High enzyme	6.5	0	0.0022	308.6	ND
		4	0.0451	15.4	Transient
	8.0	0	0.0055	125.0	ND
		4	0.1292	5.4	Transient
Low enzyme	6.5	0	ND	∞	ND
		1	0.0016	427.9	0.0126
	8.0	0	ND	∞	ND
		1	0.0073	94.6	0.0216

### Peptide mapping of Pru p 3 digests

Peptide profiling of 120 min digests was undertaken by LC–MS/MS before and after reduction and alkylation. The level of sequence coverage of the different Pru p 3 isoforms varied from 77.8 to 100% (Supplementary Information S1, Data sheet 3) with all seven isoforms identified with unique peptides (Supporting information, Fig. S6). Three of the sequences are splice variants of the LTP gene PRUPE_6G292600 identified in the *P. persica* genome sequence (A0A251NXA3, A0A251NXA4 and A0A251NXB7). All three variants have an N-terminal extension with A0A251NXA4 also having a C-terminal extension. Peptides (including ones unique to particular sequence accessions) were identified in both the extensions suggesting that translation of the longer transcripts takes place in the plant and that posttranslational processing to remove the N- and C-terminal sequences is incomplete. However, it seems likely these are minor components since the majority of the protein purified using the protocol applied here has previously been shown to have a N-terminal sequence corresponding to the fully processed protein and an intact mass corresponding to either the Pru p 3 sequence Q8H2B2^14^ or Q9LED1^15^.

Between 97–114 and 21–23 peptides ranging from 6 to 30 amino acid residues in length were identified in the reduced and non-reduced samples respectively, with good reproducibility between biological triplicates (Supporting information, Figs. S4 and S5). Post-translational modifications identified included deamidation of glutamine, asparagine, and phosphorylation of serine (Supplementary Information 2, Datasheet 1). These included peptides ^23^IAQAITCGQVS^33^ and ^17^MVVSVPIAQAITCGQV corresponding to the N-terminal extensions found in Pru p 3 sequences Q5RZZ3, A0A251NXA3, A0A251NXA4 and A0A251NXB7.  These also had phosphorylated serine residues, ^3I^AQAITCGQVS^33^ also being identified in an unmodified form in some samples. Three phosphoserines (^55^S, ^57^S, ^82^S) were identified with high confidence, in all digestion conditions, in peptides corresponding to residues 57–72 and 78–85, 80–91, 51–61 (Supplementary Information S2, Data sheet 1 and 2; Supplementary Information S3, Datasheet 1 and 2; Example extracted ion chromatograms for each are shown as Supporting information Fig. S8).

**Figure 2:**
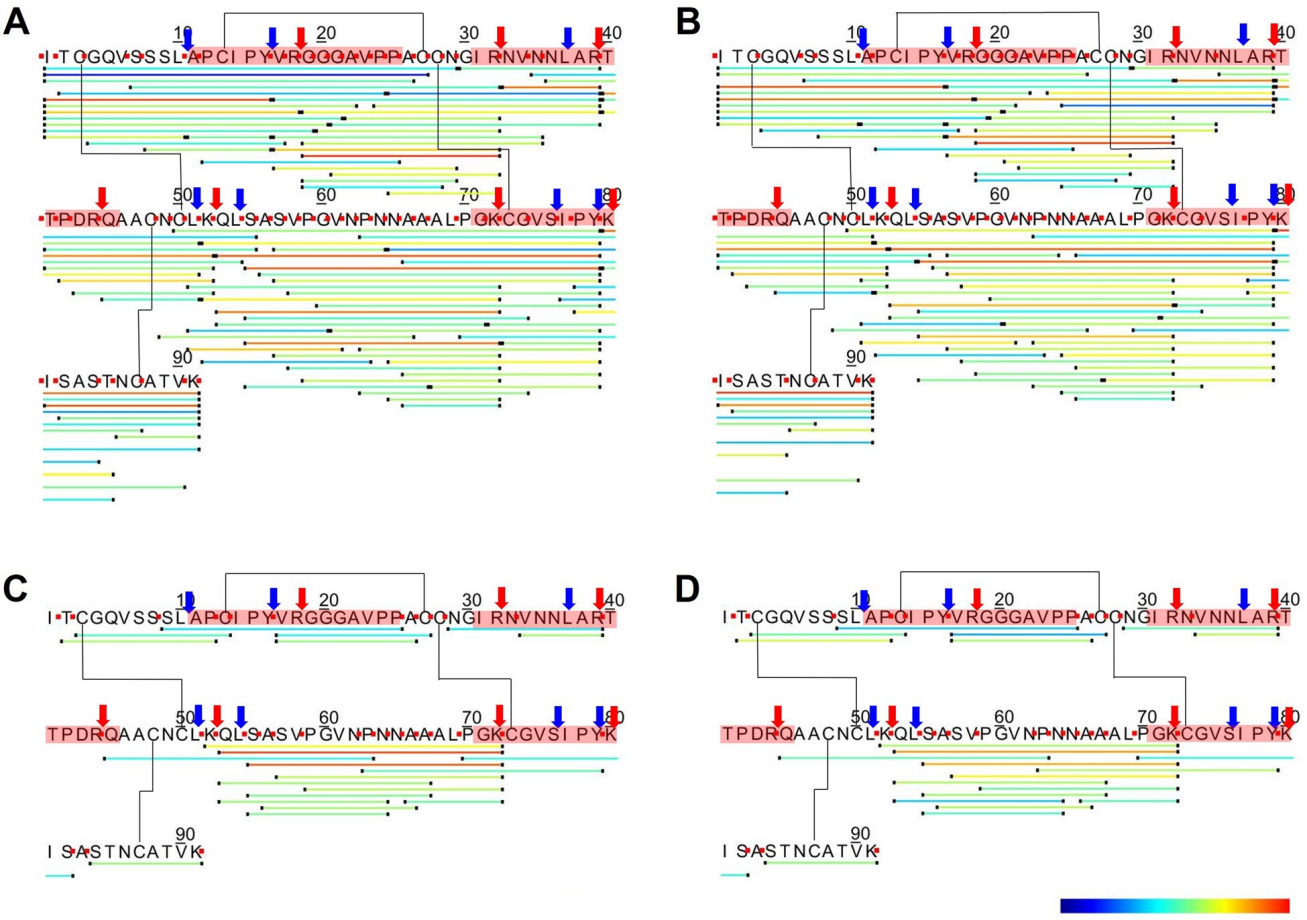
Peptide profiling of Pru p 3 after gastrointestinal digestion using the high enzyme test performed in the presence of bile salts. Samples were analysed prior to (**A**,**B**) and after reduction (**C**,**D**). Peptides were mapped onto UniProt accession: Q9LED1. The disulphide bonds are linked by black lines. Linear IgE epitopes are highlighted in pink based on García-Casado (2003). Red arrows: predicted tryptic cleavage sites; blue arrows: predicted chymotryptic cleavage sites. Colour shading represents relative abundance of peptides calculated using spectral counting with the gradient from blue to red indicating increasing abundance.

Peptides were then mapped onto the Pru p 3 sequence using accession Q9LED1 which corresponds to the isoform Pru p 3.0102 (Fig. 2; Supporting information, Fig. S5), an accession for which there is strong protein level evidence^16^ and to allow comparison with previous work^17^. Analysis of the reduced and alkylated samples gave a complete overview of all the digestion products identified and showed a complex mixture of overlapping and nested peptides. In general peptides were less abundant in the low enzyme compared to the high enzyme test and the number decreased in the duodenal (pH 6.5) compared to the intestinal (pH 8.0) digests. All seven predicted specific tryptic cleavage sites were identified in the digests although Arg44-Gln45 was cleaved poorly in the low enzyme test (Supporting information, Fig. S5). Five of the six predicted chymotryptic cleavage sites were also identified although the one spanning residues 10L-11A was less favoured. In addition, a number of non-typical cleavage sites were observed. Certain peptides dominated the digests indicating there are preferred pathways of proteolysis, such as the N-terminal peptides I1-A26 and I1-N29, peptides spanning the central region, L51-V61, C50-Y79 and S56-N64 together with the C-terminal peptides A66-K91 and N86-K91. The data showed that one IgE epitope located at residues I31-T40 was digested whilst the other two, G71-K80 and A11-G20, were partially degraded.

Since many of the dominant peptides spanned the disulphide bonds, analysis was also undertaken on non-reduced digests (Fig. 2C,D; Supporting information, Fig. S5C,D). This analysis only identified peptides spanning the Pru p 3 sequence between disulphide bridges, most of which were located between Cys50 and Cys73 and included the dominant peptide Q53-K72 together with the fragment S55-K72 which was dominant only in the high enzyme test (Fig. 2C,D; Supporting information, Fig. S5C,D).

### Immunoreactivity of Pru p 3 digests

**Figure 3:**
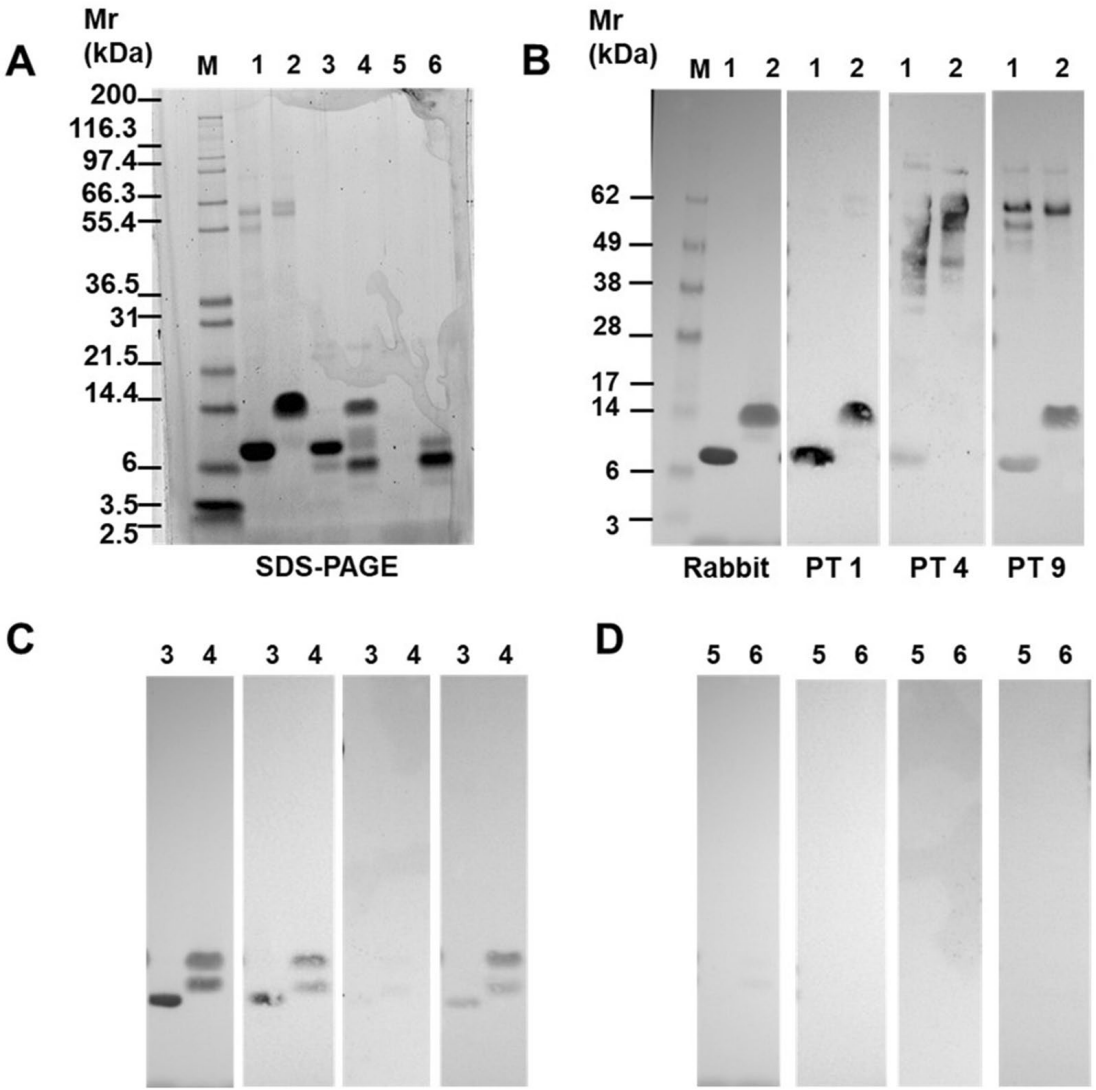
SDS-PAGE (**A**) and immunoblot (**B**–**D**) of Pru p 3 digests after gastrointestinal digestion at pH 8.0 using the high enzyme test performed in the absence and presence of bile salts. Lanes were as follows: M—molecular weight makers; lanes 1, 3, 5—samples analysed following reduction; lanes 2,4,6—samples analysed without reduction. Lanes 1, 2—undigested Pru p 3; lanes 3–6—gastrointestinal digests performed in the absence (lanes 3, 4) and presence (lanes 5, 6) of bile salts. Rabbit—immunoblot developed using anti-Pru p 3 antibody preparation: PT—patient no. (**B**) Immunoblots of undigested Pru p 3; (**C**) immunoblots of gastrointestinal digests of Pru p 3 performed in the absence of bile salts; (**D**) immunoblots of gastrointestinal digests of Pru p 3 performed in the presence of bile salts.

Since the MS peptide profiling indicated that the major IgE-epitopes were largely degraded, the impact of digestion on the immunoreactivity of Pru p 3 was assessed by immunoblotting and immunoassay. Initial analysis using an anti-Pru p 3 antibody preparation raised in rabbits showed that reducing conditions had no effect on immunoreactivity (Fig. 3 B Rabbit lane 1) with both the Mr ~ 9 kDa reduced Pru p 3 and the Mr ~ 14 kDa non-reduced Pru p 3 being equally well recognised. Analysis of the 120 min gastrointestinal digest performed using the high enzyme test at pH 8.0 confirmed that Pru p 3 was partially digested with residual intact Pru p 3 evident as a polypeptide of Mr ~ 14 kDa. This was accompanied by a polypeptide of Mr ~ 7 kDa, a faintly staining, poorly resolved band of Mr ~ 9–11 kDa and a very faint band of Mr ~ 4.5 kDa. Only the residual intact Pru p 3 and a Mr ~ 11 kDa band retained their immunoreactivity (Fig. 3C Rabbit lane 4). Analysis of the reduced digest performed in the presence of 4 mM bile salts showed the protein was completely digested but when analysed under non reducing conditions a Mr ~ 7 kDa band predominated which was accompanied by two others of Mr ~ 9.0 and 4.5 kDa (Fig. 3A lane 6). However, virtually none of the residual Pru p 3 was immunoreactive with only very faintly staining polypeptides evident when analysed under non-reducing conditions (Fig. 3D Rabbit lane 6).

A similar pattern of reactivity was also observed with serum IgE from peach allergic patients although there was some variability between patients with some individuals also reacting with the Mr 55 and 60 kDa contaminants (Fig. 3 PT1, 4, 9; Supporting information, Fig. S9). Serum IgE from four patients (patients 3–6, Supporting Information, Fig. S9) showed only weak recognition of Pru p 3 and only under reducing conditions. The remaining patients had serum IgE that recognised the protein irrespective of its reduction status although some showed a lower level of reactivity towards the non-reduced Pru p 3 (patients 2, 7, 8, 11 and 12) whilst one patient appeared to recognise the non-reduced protein more strongly (patient 9) (Fig. 3B; Supporting information, Fig. S9A). There was no clear correlation between the specific IgE level to Pru p 3 determined by ImmunoCAP, the severity of symptoms experienced by the patients and IgE reactivity on immunoblotting (Supporting information, Table 1).

In general, the IgE-binding capacity of Pru p 3 was reduced by digestion (Fig. 3C; Supporting Information, Fig. S9B) and displayed two different patterns. In the first of these IgE from patients 1, 2, 7–13 bound digested Pru p 3 in a similar way to the rabbit anti-Pru p 3 antibody preparation recognising the large digestion fragment of Mr ~ 11.0 kDa as well as intact Pru p 3 analysed under non-reducing conditions. In a second group of patients (nos 3–6) who exhibited very low reactivity towards Pru p 3 on immunoblots, lost all IgE-reactivity to digested Pru p 3. Digestion in the presence of bile salts completely abolished the IgE reactivity of Pru p 3 for all patients (Fig. 3 D; Supporting Information, Fig. S9C).

**Figure 4:**
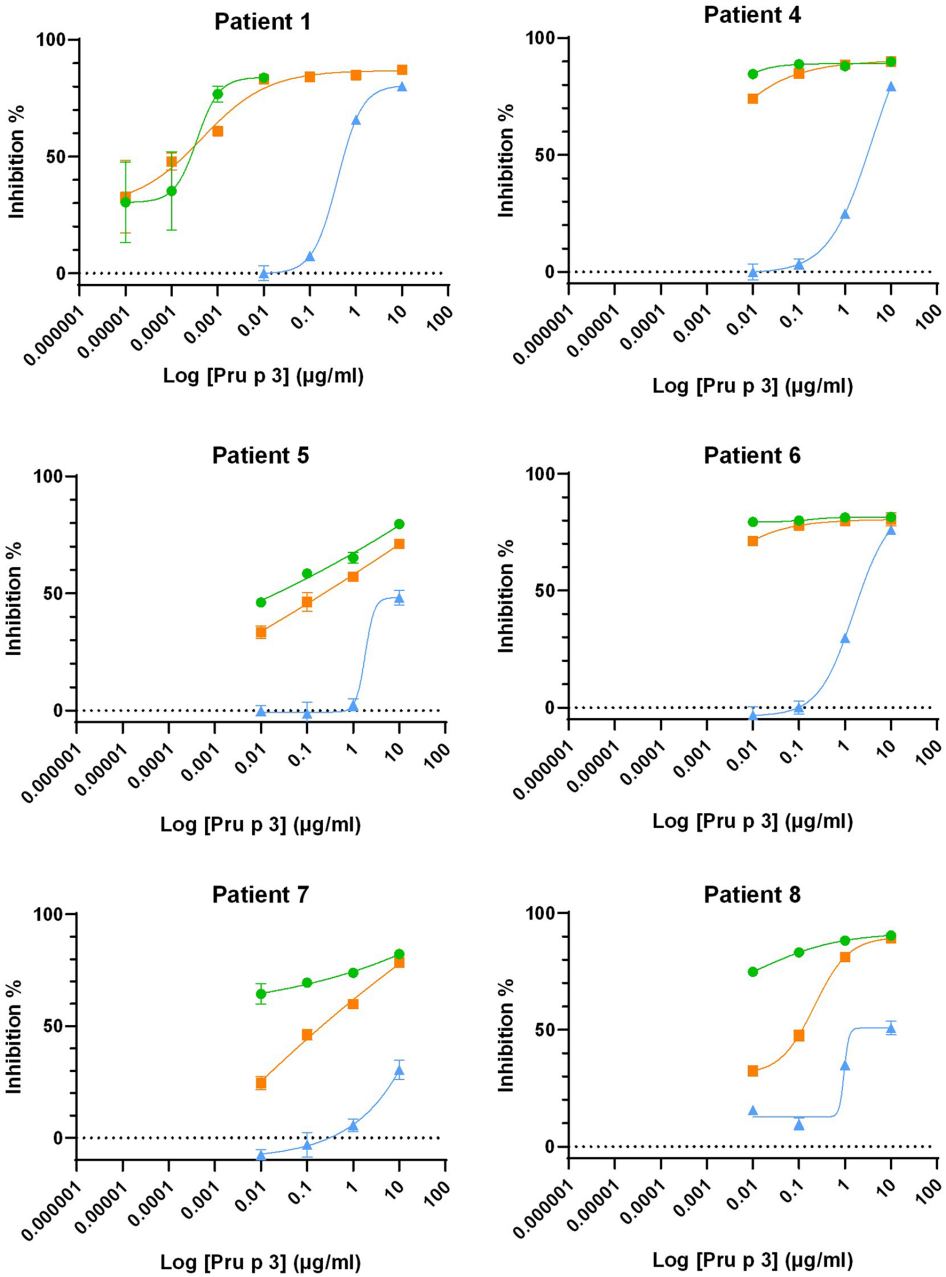
Effect of gastrointestinal digestion on the IgE-reactivity of Pru p 3 determined by inhibition ELISA. Undigested Pru p 3: ; gastrointestinal digest of Pru p 3 performed in the absence of bile salts: ; gastrointestinal digest of Pru p 3 performed in the presence of bile salts: . Curves were fitted using a four-parameter logistic model.

Additional confirmatory analysis of IgE reactivity was undertaken using an inhibition ELISA for six patients where sufficient serum was available (Fig. 4) from which the protein concentration able to inhibit binding by 50% could be calculated (Table 2). Intact Pru p 3 showed the highest IgE binding capacity with IC50 values of ≤ 0.01 μg/mL of Pru p 3 except for patient 6 for whom it was 0.17 μg/mL. Digests performed in the absence of bile salts exhibited either similar (Patient 1, 4, 5 and 6) or slightly lower (Patient 7 and 8) IgE binding capability with IC50 values similar to intact Pru p 3. In contrast, digestion in the presence of bile salts drastically reduced IgE-binding capacity with IC50 values increasing by between 100 and 1000-fold (Table 2).

**Table 2:** Effect of in vitro gastrointestinal digestion on Pru p 3 IC50 values. IC50 values were calculated from ELISA inhibition curves. (~) indicates estimated values as the curve was not properly fitted.

Patient no	Pru p 3 sample IC50 (µg/mL)		
	Intact	Gastrointestinal digest	Gastrointestinal digest in the presence of 4 mM bile salts
P1	< 0.01	< 0.01	0.40
P4	< 0.01	< 0.01	3.89
P5	~ 0.02	~ 0.25	> 10
P6	0.17	< 0.01	1.57
P7	< 0.01	< 0.01	> 10
P8	0.01	0.21	> 10

## Discussion

This study has demonstrated that bile salts accelerate the rate of trypsin and chymotrypsin catalysed cleavage of Pru p 3. Digestion performed without these surfactants left Pru p 3 either undigested (low enzyme test at pH 6.5 and 8.0) or degraded with a half-life ranging from just over 300 min at pH 6.5 to 125 min in the high enzyme test at pH 8.0. Even in the presence of bile salts digestion of Pru p 3 is much slower than many other food proteins such as the highly digestible cow’s milk caseins^21,23^. Such data suggest that individuals with either impaired intestinal digestion or bile secretion (such as patients with choleostasis or infants)^24,25^ would not be able to digest Pru p 3 and that the protein would persist for many hours in the gastrointestinal tract in an intact form.

Digestion resulted in the formation of large fragments which, depending on the in vitro digestion conditions, persisted. And as observed by others a major tryptic cleavage site is found at Y79 which is responsible for generation of a Mr ~ 7 kDa fragment^15–17^. Previous studies showed pre-loading of Pru p 3 with linoleic acid enhanced Pru p 3 digestibility at pH 6.8 (duodenal conditions) even in the presence of bile salts^17^ and competition binding studies indicated that bile salts were unable to displace the fluorescent lipid cis-parinaric acid^26^. It maybe the bile salts can bind to the unoccupied calyx and would account for the preferred chymotryptic cleavage site at Y79 which is more mobile and is made more accessible to chymotrypsin when a ligand is present in the lipid binding tunnel of Pru p 3^17^. Intriguingly we have found evidence of phosphorylation of Pru p 3 serine residues, including the serine residue in the consensus calmodulin binding region at the C-terminus of the protein which modulates cellular uptake of viral particles facilitated by maize LTP^27^. It has also been reported previously that every serine residue in petunia LTP has the capacity to be phosphorylated in vitro^28^. It maybe that bile salt binding to Pru p 3 follows a pathway like that of serum albumin, where initial specific binding is followed by an opening up of the protein structure accompanied by unfolding which allows further bile salt binding, all mediated through hydrophobic interactions^29^.

Many of the peptide digestion products were held together as larger entities of Mr ~ 6 kDa on SDS-PAGE by intramolecular disulphide bonds. It is likely this fragment corresponds to the large 5.7 kDa fragment previously identified by mass spectrometry, as all the constituent peptide fragments identified by MS only appeared in the reduced and alkylated sample. However, although we were not able to confirm the IgE reactivity of this fragment by SDS-PAGE and immunoblotting, the digest retained some residual IgE binding by ELISA suggesting that this was the case. This confirms previous observations that although all three major IgE epitopes in Pru p 3 were digested, the intramolecular disulphide bonds allow sufficient three dimensional structure to be retained such that some IgE binding is retained^16^. This is also consistent with the observations that conformational epitopes dominated the allergenic potential of Pru p 3 and that after disulphide bond reduction and alkylation, IgE reactivity is decreased^18^.

This report indicates that it is important to consider inclusion of bile salts in in vitro digestion tests used for allergenicity risk assessment^30,31^ since they may enhance digestion of proteins that might otherwise be considered highly resistant. This study also demonstrates the importance of taking the role of intramolecular disulphide bonds into account in the analysis of protein digestion products. Further studies are needed to understand whether the observations reported here can be generalised to other lipid-binding proteins and understand the molecular basis for the interaction of proteins with bile salts that alters digestibility. It is also critical to understand the molecular mechanisms underpinning the impact of bile salts on rates and pathways of protein digestion if effective in silico models of digestion are to be built in future.

## Methods

### Enzymes, proteins and other reagents

Chemicals were of analytical reagent grade and purchased from Sigma-Aldrich (Gillingham, UK) unless otherwise stated. Digestion enzymes were purchased as freeze-dried powder and the protease activities were checked prior to use as follows: pepsin was from porcine gastric mucosa (1799 U/mg determined using haemoglobin as substrate^32^; trypsin was from porcine pancreatic mucosa (1959 U/mg of protein determined using benzoylarginine ethyl ester as the substrate^33^; bovine pancreatic α-chymotrypsin (52 U/mg of protein determined using benzoyltyrosine ethyl ester as the substrate^34^. Other biochemicals for in vitro digestion were soybean Bowman-Birk trypsin-chymotrypsin inhibitor, sodium taurocholate (≥ 95% purity by thin layer chromatography [TLC]), and sodium glycodeoxycholate (≥ 97% purity by TLC). Ultrapure water was purified using a Milli-Q ultrapure water system (Millipore, Darmstadt, Germany).

### Pru p 3 purification

Pru p 3 was purified from peach skin as described previously by a combination of ammonium sulphate fractionation and cation exchange chromatography based on published procedures^14^. Peach peel was flash-frozen in liquid nitrogen, ground and the resulting powder extracted add ~ 1:1 (w:v) ratio in 50 mM sodium phosphate buffer containing 2 mM ethylenediaminetetraacetic acid (EDTA), 20 mM sodium diethyldithiocarbamate, 3% (w:v) polyvinylpolypyrrolidone, 0.02% (w:v) NaN_3_, pH 7.0 by stirring at ambient temperature for 1 h. The resulting suspension was clarified by centrifugation at 1700×*g* for 30 min at 10 °C, the supernatant removed, and ammonium sulphate fractionation used to collect the protein that precipitated between 40 and 95% saturation by centrifugation at 1700×*g* for 30 min at 10 °C. The resulting pellet was resuspended in water (1:1; w:v) and salt removed by buffer exchange against deionized water before adjusting the pH to 6 after adding 20 mM 4-morpholineethanesulfonic acid (MES) and 3 mM NaN_3_. Finally, the dialysate was clarified by centrifugation at 22,100×*g* for 30 min at 10 °C, the supernatant removed and applied to a XK 26/20 column (200 × 26 mm, Cytiva, Amersham, UK) packed with SP-Sepharose high Performance (73.3 mL, Cytiva, Amersham, UK) attached to an ÄKTA system (Cytiva, Amersham, UK) equilibrated with 20 mM MES pH 6.0. Bound protein was eluted with a linear salt gradient (buffer A: 20 mM MES, pH 6.0; buffer B: 0.6 M NaCl; 0–100% (v/v) over 30 column volumes using a flow rate of 5 mL/min. The eluate was monitored for protein by UV absorbance at 280 and 220 nm and protein- containing fractions analysed by SDS-PAGE. Those fractions containing Mr ~ 9 kDa proteins were pooled and stored at − 20 °C. Protein was adjudged > 95% pure by SDS-PAGE analysis with minor contaminants observed of Mr ~ 55 and 60 kDa.

### Patient sera

Peach allergic patients were recruited from Hospital General Universitario de Alicante (Valencia) in accordance with the protocol approved by the hospital ethics committee ISABIAL (Instituto de Investigación Sanitaria y Biomédica de Alicante), PI2020-005. All procedures were performed in compliance with that approval and relevant laws and institutional guidelines and informed consent was obtained from all human subjects. IgE-mediated food allergy was confirmed by a combination of assessment of clinical manifestations, skin prick test and serum specific IgE to Pru p 3. Thirteen patients (seven females and six males, ranging from 22 to 53 years old) were selected with Pru p 3 specific sIgE levels ≥ 2.5 ISU of IgE/L (Supporting information, Table S1).

### In vitro gastrointestinal digestion

In vitro gastric digestion was performed using a low pepsin test^21,35^. Briefly, Pru p 3 was dissolved in simulated gastric fluid (SGF, 150 mM NaCl, pH 2.5) to a concentration of 1 mg/mL, warmed to 37 °C and the pH was adjusted to 2.5 by addition 0.1 M HCl. An equal volume of prewarmed pepsin in SGF (pH 2.5) was added to give 165 U of pepsin per mg of Pru p 3 in the final digestion mix which comprised 3 mg of Pru p 3 and 0.28 mg of pepsin. The digestion was stopped by placing the sample on ice and raising the pH to 7.5 using 0.1 M ammonium bicarbonate after 10 min.

The gastric digest was used immediately for the intestinal digestion and warmed to 37 °C before re-adjusting the pH to either 6.5 or 8.0 using 0.1 M NaOH^36^. One hundred μL of the digest solution was added to each well of a microtitration plate (Corning, Loughborough, UK) and warmed to 37 °C in a shaking water bath (170 rpm) for 10 min. Trypsin (103.5 U/mL or 10.4 U/mL), chymotrypsin (1.2 U/mL or 0.12 U/mL) and bile salts were added to chilled simulated intestinal fluid (SIF, 150 mM NaCl, pH 6.5 or 8.0) immediately prior to use, warmed to 37 °C for 10 min and 20 μL/well added to the microtitration plate. The final concentration of reagents dissolved in 26.1 mM bis–Tris buffer at either pH 6.5 or 8.0 were as follows:

1. High enzyme test: 4 mM sodium taurocholate, 4 mM sodium glycodeoxycholate, 0.4 U/mg of Pru p 3 of bovine α-chymotrypsin, 34.5 U/mg of Pru p 3 of porcine trypsin.
2. Low enzyme test: 1 mM sodium taurocholate, 1 mM sodium glycodeoxycholate, 0.04 U/mg of Pru p 3 of bovine α-chymotrypsin, 3.5 U/mg of Pru p 3 of porcine trypsin.

Digestion was quenched by transferring 110 μL/well of the digest into a second microtitration plate containing 10 μL/well of a two-fold excess of Bowman-Birk trypsin-chymotrypsin inhibitor from soybean (Sigma) dissolved in 150 mM NaCl pH 6.5 at either 0.5 mg/mL (high enzyme test) or 0.1 mg/mL (low enzyme test). Intestinal undigested control (IU) comprised 0.5 mg/mL Pru p 3 in SIF. Digest samples were either analysed immediately or plates sealed using parafilm (Sigma-Aldrich, Gillingham, UK) and stored at − 20 °C until required.

### SDS-PAGE analysis

Samples (55 μL) were prepared for SDS-PAGE by adding 20 μL NuPAGE (LDS) buffer (Thermo Scientific, Hertfordshire, UK), and 5 μL of either 0.5 M dithiothreitol (DTT) (reduced samples) or ultrapure water (non-reduced samples). Reduced samples were heated to 100 °C for 5 min and cooled to ambient temperature prior to loading to 4–12% Bis–Tris gels in a NuPAGE system (Invitrogen, Thermo-Fisher Scientific, Paisley, UK). Mark 12 Unstained Standard (Invitrogen) was used as molecular weight markers for protein-stained gels and SeeBlue pre-stained markers (Invitrogen, Loughborough, UK) were used for gels to be electroblotted. Proteins were separated according to the manufacturer’s instructions using 200 V, 350 mA and 100 W for 35 min. Gels were fixed in 50% (v/v) methanol, 10% (v/v) acetic acid and after 1 h rinsed three times for 5 min each in deionised water before staining with Coomassie G-250 stain (SimplyBlue, Invitrogen). The gel was de-stained by rinsing with MilliQ water and imaged using a Typhoon gel Scanner (Amersham, UK).

### Densitometry and kinetics analysis of SDS-PAGE gels

The semi-quantitative analysis of the band intensity were carried out based on a previously described method^35,37^. Statistical analysis was performed using Microsoft Excel and the R-Studio^38^. A test of significance was performed between the relative band intensity of control “IU” and “120 min” to define if Pru p 3 was completely resistant and was omitted from the kinetic analysis, using a two-tailed Student’s t-test with 0.05 level of significance. Models of the kinetics of protein hydrolysis were built based on an exponential curve fit built using R (https://zenodo.org/record/6402699#.Ykgye-fMJm8) using the Levenberg–Marquardt Algorithms for computing damped least-squares and the goodness of fit assessed using the residual standard error (RSE).

### Reverse phase-high performance liquid chromatography

Digest samples (65 μL) were applied to Jupiter^®^ C18 RP column, 4.6 mm id × 250 mm, 5 µm particle size (Phenomenex, Torrance, CA, USA) attached to an Agilent HPLC system (Agilent, Cheshire, UK) with a diode array detector modified from the method previously described by Mandalari et al.^39^. Samples were eluted using 0.1% (v/v) trifluoroacetic acid (TFA) in purified water as solvent A and 0.1% (w/v) TFA in acetonitrile as solvent B (gradient of 1 mL/min held at 5% solvent B for 5 min followed by a linear gradient to 60% solvent B for 60 min).

### LC–MS/MS analysis

Peptide profiling of digests (500–5000 Da) with and without reduction was performed using liquid chromatography with tandem mass spectrometry (LC–MS/MS). Samples were reduced by addition of 330 µL of 50 mM ammonium bicarbonate and 40 µL of 50 mM DTT to 25 µL of digest and heated to 80 °C for 10 min. Samples were subsequently alkylated by addition of 45 µL of 150 mM iodoacetamide and incubated at ambient temperature for 30 min in the dark. Samples were de-salted by solid-phase extraction using Oasis HLB 96-well µElution Plate (Waters, Wilmslow, UK) and diluted to the equivalent of 30 μg Pru p 3/mL. A volume of 5 µL was injected onto a nanoAcquity UPLC system fitted to a Xevo G2-SX QToF mass spectrometer (Waters, Wilmslow, UK) equipped with a NanoLockSpray dual electrospray ion source was used. Samples were concentrated on a nanoEaseTM M/Z trap column (Symmtery C18, 100 Å, 5 µm, 180 µm × 20 mm; Waters, Wilmslow, UK) and separated on a nanoEase M/Z Peptide BEH C18 Column (1.7 µm, 75 µm internal diameter × 200 µm length, 130 Å pore size; Waters, Wilmslow, UK) with a column temperature of 35 °C. A flow rate of 0.5 µL/min using 0.1% (v/v) formic acid in HPLC grade water (solvent A) and 0.1% (v/v) formic acid in acetonitrile (solvent B) was utilised for peptide separation. The elution gradient first rose from 5 to 35% solvent B over 30 min, then up to 85% solvent B over 5 min before column re-equilibration. The mass spectra were obtained in positive ion electrospray mode using the *m/z* range 50–2000. A lock spray of LeuEnk (556.2771 Da) was set to optimise the mass measurement every 60 s, with 0.5-s inter-scan time.

The MS/MS spectra were analysed using Progenesis QI (Waters, Wilmslow, UK) with a database composed Pru p 3 Uniprot accessions P81402, Q5RZZ3, A0A251NXA4 and A0A251NXB7 together with Uniprot sequences of the proteases and proteinase inhibitor. No specific enzymatic cleavage was specified, and the possible modifications searched were serine phosphorylation, methionine oxidation, and deamidation of glutamine or aspartic acid. Carbamidomethyl cysteine was set as fixed modification for reduced-alkylated samples. Peptides identified with score higher than 5 were retained with an evaluated false discovery rate of less than 1%. The MS data have been deposited in the PRoteomics IDEntifications (PRIDE) Archive database^40^, with a dataset identifier of PXD037801.

### Immunoblotting

Unstained SDS-PAGE gels were electroblotted onto a nitrocellulose membrane using a Trans-blot SD semi-dry transfer cell (Biorad, Hertfordshire, UK) as previously described^41^. Blots were developed as previously described^42^ using either a polyclonal rabbit anti-Pru p 3 antibody preparation or serum containing IgE from peach allergic patients followed by horseradish peroxidase (HRP)-conjugated mouse anti-rabbit IgG or anti-human IgE antibody respectively. The bound antibodies were detected using a chemiluminescent substrate (Pierce SuperSignal™ West Dura Extended Duration Substrate). The membranes were imaged with equal exposure time using GeneGnome (Syngene, Cambridgeshire, UK).

### Immunoassay

Inhibition ELISA was carried as previously described^43^ except 96 well plates were coated with purified intact Pru p 3 (10 µg/mL) diluted in PBS (pH 7.4) overnight at 4 °C. Human serum samples were diluted 1:20 (v:v) in 0.1% (w/v) bovine serum albumin (BSA), 0.05% (v/v) Tween-20 in 0.01 M PBS, pH 7.4 (PBST apart from those from patient 1 which were diluted 1:100 (v:v)). Diluted serum (50 μL) was incubated at 37 °C for 1 h with 50 µL of either intact Pru p 3 or Pru p 3 digest diluted in 1:50 to 1:50,000,000 (v:v) in PBST to give the equivalent of 0.00001 to 10 µg/mL of intact Pru p 3 with 0.01 M PBS buffer serving as a negative control. The mixture (100 µL/well) was added to the microtitration plates and incubated overnight at 4 °C. After washing 100 μL/well of mouse anti-human IgE antibody conjugated to horseradish peroxidase (HRP) (Southern Biotech, Cambridge, UK) (diluted 1:5000, v:v) was added to the plate and incubated for 1 h at 37 °C. After further washing steps 3,3′,5,5′-tetramethylbenzidine (ThermoFisher Scientific, Hertfordshire, UK) was added (100 µL/well) and the reaction was stopped by adding 100 µL/well 1 M HCl and the absorbance at 450 nm of each well determined. Results were analysed with GraphPad Prism 9 adjusting the curve to a sigmoidal model from which IC50 values were obtained.

### Supplementary Information

Supplementary Information 1.

Supplementary Information 2.

Supplementary Information 3.

Supplementary Information 4.

## Data Availability

The data that support the findings of this study are available from the corresponding author upon reasonable request. The datasets generated and/or analysed during the current study are available in the PRIDE partner repository, with the dataset identifier PXD037801. Reviewer account details are as follows: Username—reviewer_pxd037801@ebi.ac.uk; Password: fF7IgcOM.
