# Supplementary material for: Bile salts enhance the susceptibility of the peach allergenic lipid transfer protein, Pru p 3, to in vitro gastrointestinal proteolysis
Source: Sci Rep. 2023 Sep 13;13:15155. doi: 10.1038/s41598-023-39599-0 (PMC10499906; doi:10.1038/s41598-023-39599-0)
Supplement: Supplementary file 1 — Supplementary Information 1. [file 41598_2023_39599_MOESM1_ESM.docx]

**Supporting information**

**Bile Salts Enhance the Susceptibility of the Allergenic Lipid Transfer Protein from Peach, Pru p 3 to *in vitro* gastrointestinal proteolysis**

Kai Wang^1^, Judit Gali-Moya^1^, María Ruano-Zaragoza^2^, Kathleen Cain^3^, Giovanni D'Auria^4^, Daly^1^, Perdita Barran^3^, Rene Crevel^1,5^, Clare Mills^1,6*^

*^1^Institute of Inflammation and Repair, Manchester Institute of Biotechnology, 131 Princess Street, Manchester, M1 7DN, UK*

*^2^Hospital Clinic of Barcelona, Barcelona, 08036, Spain*

*^3^Department of Chemistry, Manchester Institute of Biotechnology, 131 Princess Street, Manchester, M1 7DN, UK*

*^4^Department of Agricultural Sciences, University of Naples Federico II, Portici, Italy.*

*^5^René Crevel Consulting Ltd, 3 Woodlands Close, Cople, Bedford MK44 3UE, UK*

*6School of Biosciences and Medicine, The University of Surrey, Guildford, Surrey, GU2 7XH, UK*

*Correspondence should be sent to Professor Clare Mills clare.mills@manchester.ac.uk

**Figure S1.** **SDS-PAGE analysis of** **Pru p 3 subjecting to *in vitro* intestinal digestion with and without bile salts.** The digests of Pru p 3 after 10 min gastric digestion at pH 2.5 was selected for the intestinal digestion. A, C: low enzyme at pH 6.5; B, D: low enzyme, at pH 8.0; ‘IU’ is the control without enzyme. Digestions were performed in the absence or presence of 1 mM bile salts.

**
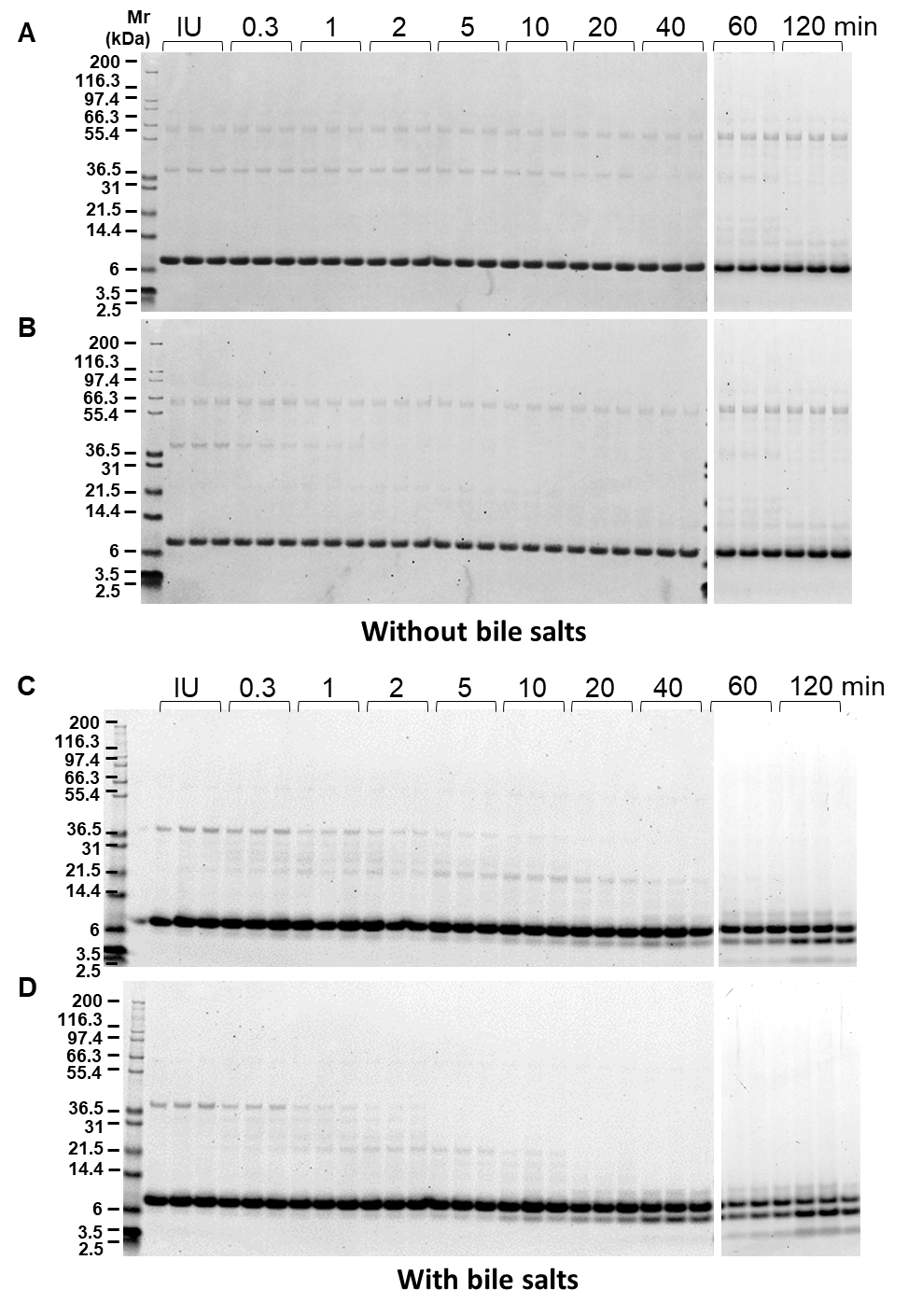
**

**Figure. S2. Reverse phase HPLC analysis of Pru p 3 subjecting to *in vitro* intestinal digestion without (Panel A-D) and with bile salts (Panel E-H).** Panel A, E: high enzyme, pH 6.5; panel B, F: high enzyme, pH 8.0; panel C, G: low enzyme, pH 6.5; panel D, H: low enzyme, pH 8.0. Red lines - IU control; black lines - 5 min; blue lines - 40 min; pink lines - 120 min.


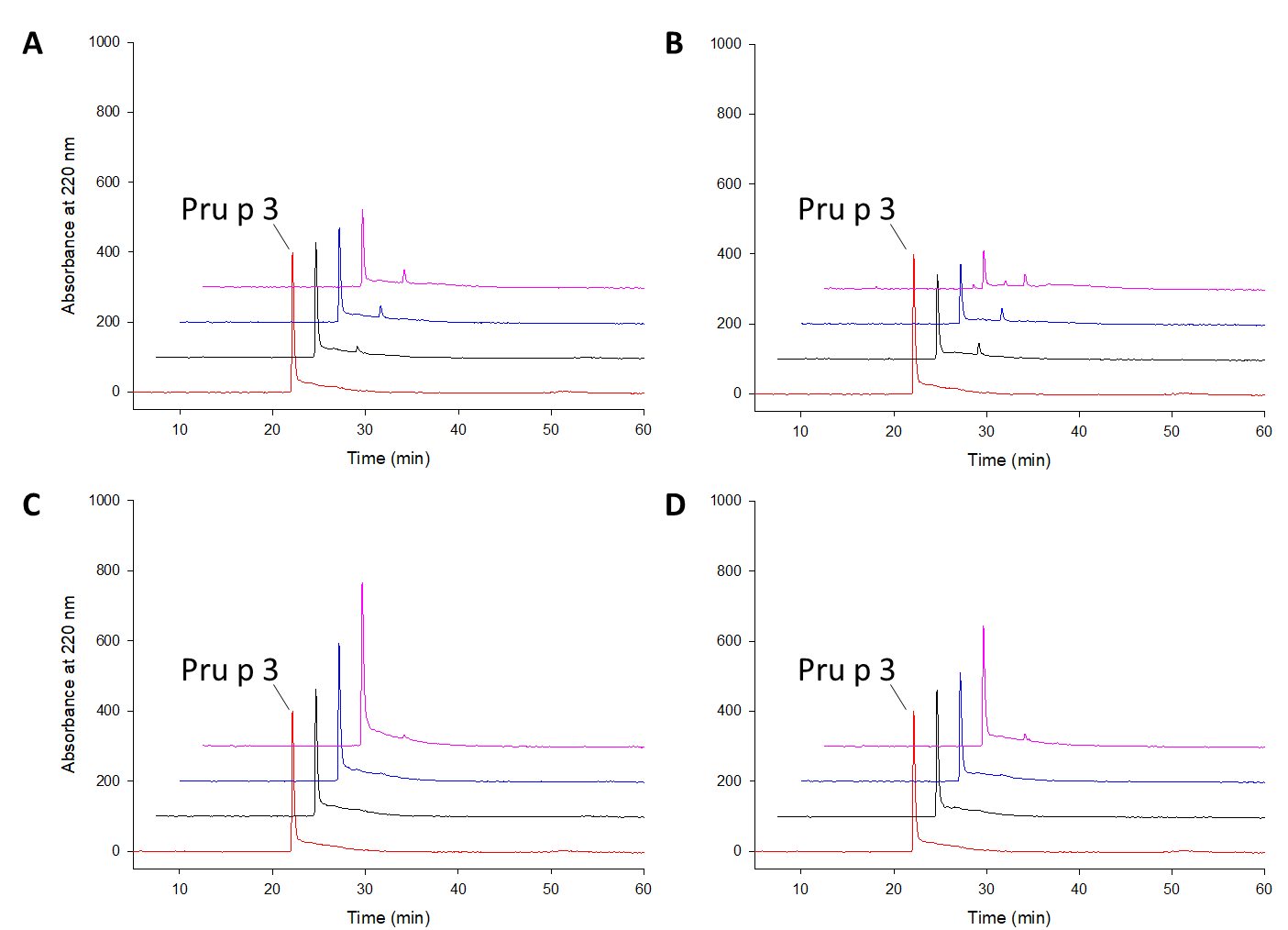


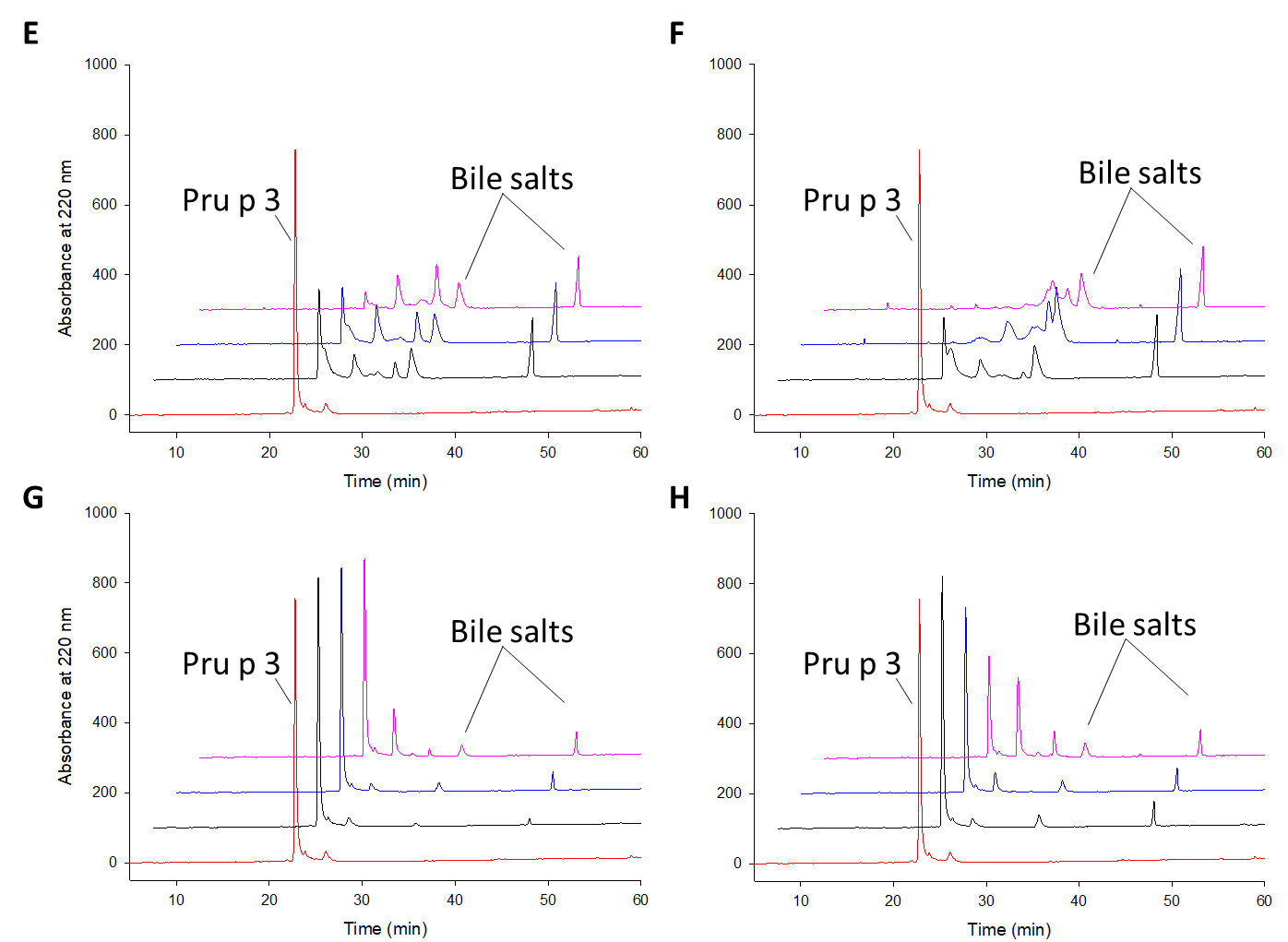


**Figure S3. Venn diagram of peptides identified corresponding to Pru p 3 across triplicates of digests in the presence of bile salts, non-reduced.**

Uniprot accessions were: Q5RZZ3; Q9LED1; P81402; A0A251NXA4; A0A251NXB3; Q8H2B2; A0A251NXB7

A - high enzyme at pH 6.5; B - high enzyme at pH 8.0; C - low enzyme at pH 6.5; D - low enzyme at pH 8.0.

**
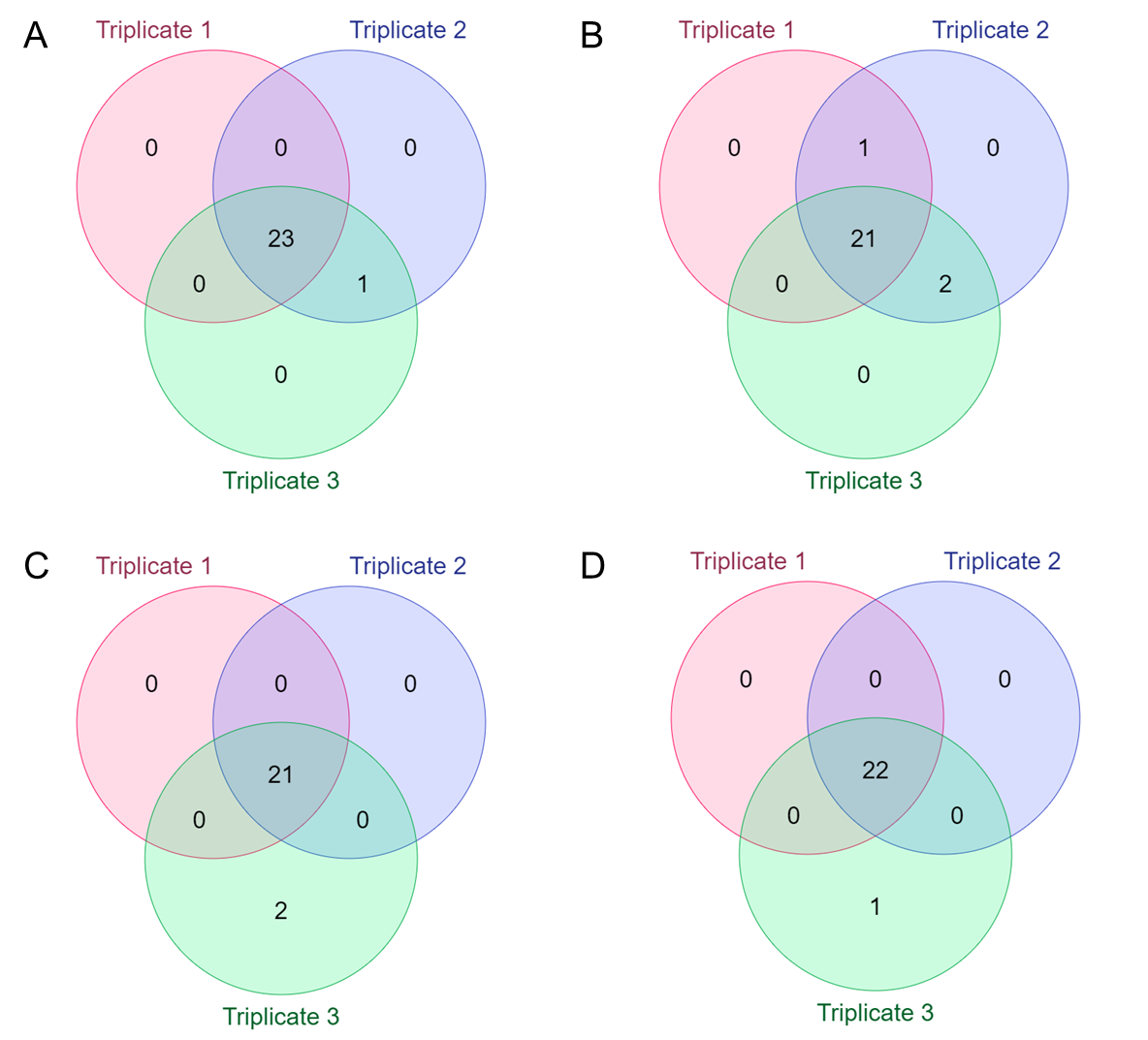
**

**Figure S4. Figure S3. Venn diagram of peptides identified corresponding to Pru p 3 across triplicates of digests in the presence of bile salts, reduced.**

Uniprot accessions were: Q5RZZ3; Q9LED1; P81402; A0A251NXA4; A0A251NXB3; Q8H2B2; A0A251NXB7

A - high enzyme at pH 6.5; B - high enzyme at pH 8.0; C - low enzyme at pH 6.5; D - low enzyme at pH 8.0.


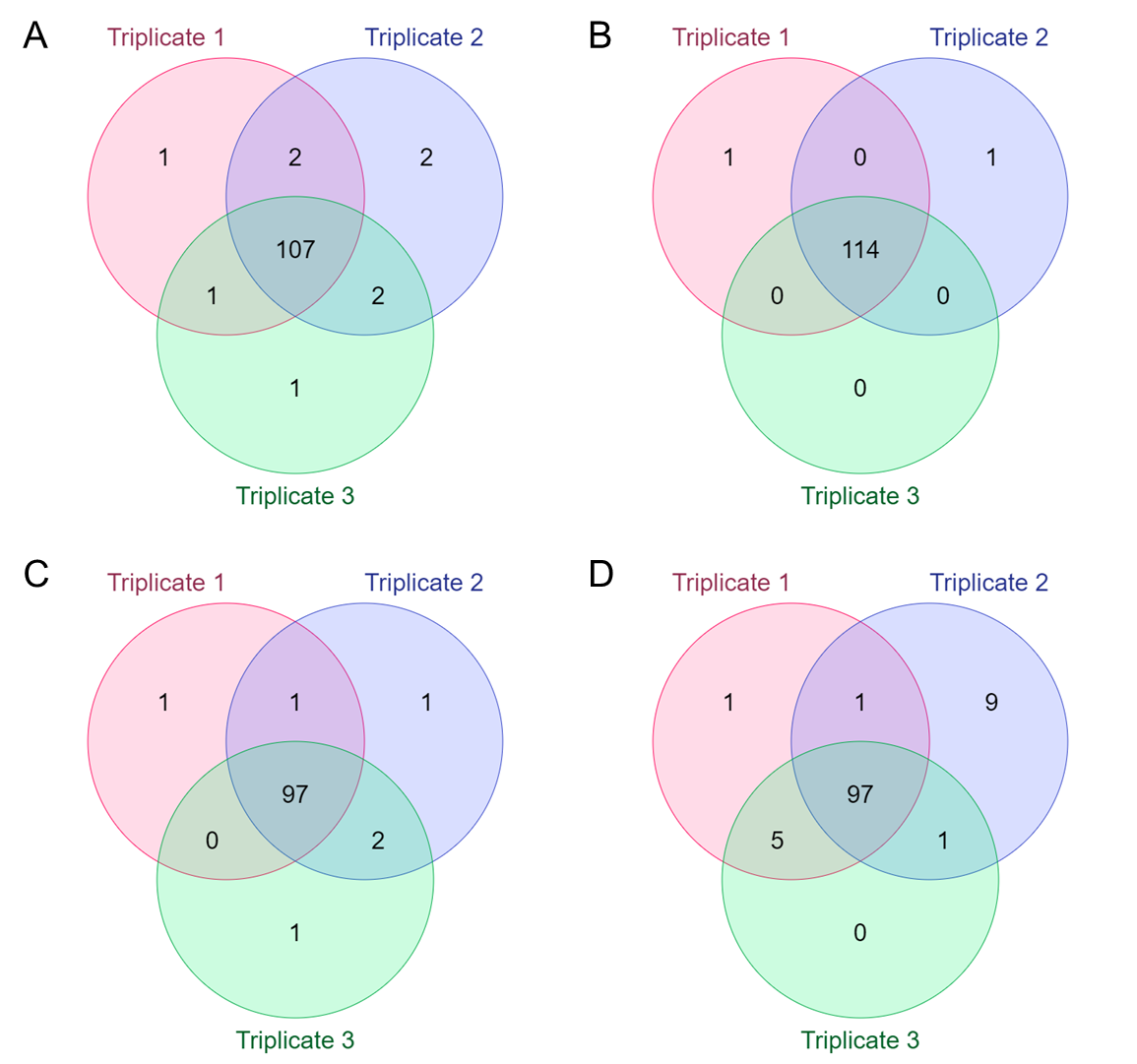


**Figure S5. Peptide mapping of the digests of Pru p 3 (UniProt accession: Q9LED1) after gastrointestinal digestion in low enzyme level with bile salts. Samples were injected under non-reducing (Panel A, B) and reducing (Panel C, D) condition.** The disulphide bonds are linked by black lines. Linear IgE epitopes are listed in pink based on García-Casado (2003). In Panel B, predicted trypsin cleavage sites are marked by red arrows and chymotrypsin cleavage sites by blue arrows. Colour shading represents relative abundance of peptides calculated using spectral counting with the gradient from blue to red indicating increasing abundance.

**
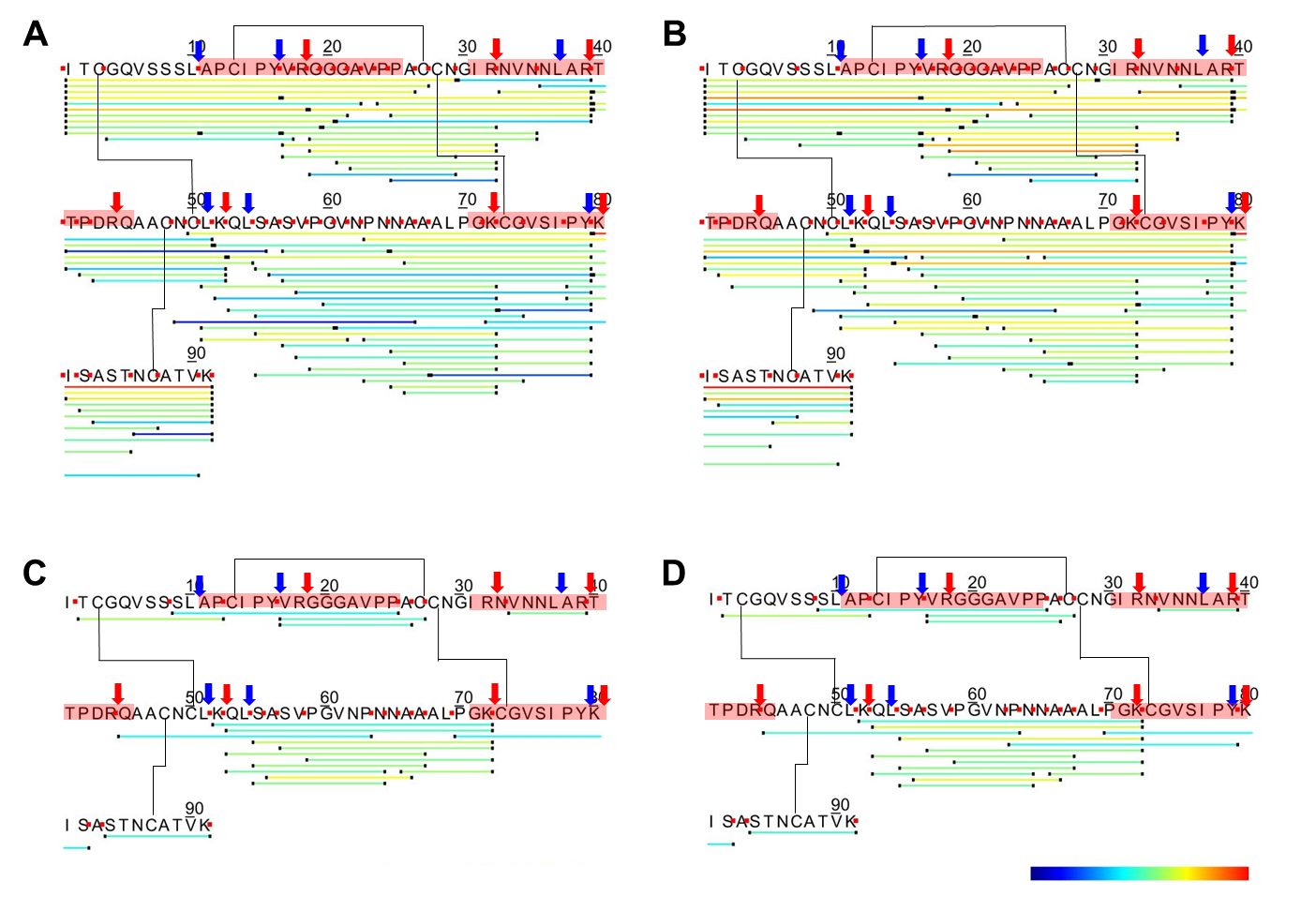
**

**Figure S6. Peptide sequence alignment of Pru p 3 isoforms and the unique peptides used to identify the isoforms from MS data (reduced).** Sequences were aligned using Clustal Omega and the unique peptides are indicated in black for UniProt accession Q8H2B2, green for P81402, blue for A0A251NXB7, red for Q5RZZ3 and Q9LED1, purple for A0A251NXA3 and A0A251NXA4, and gold for A0A251NXA4.

**
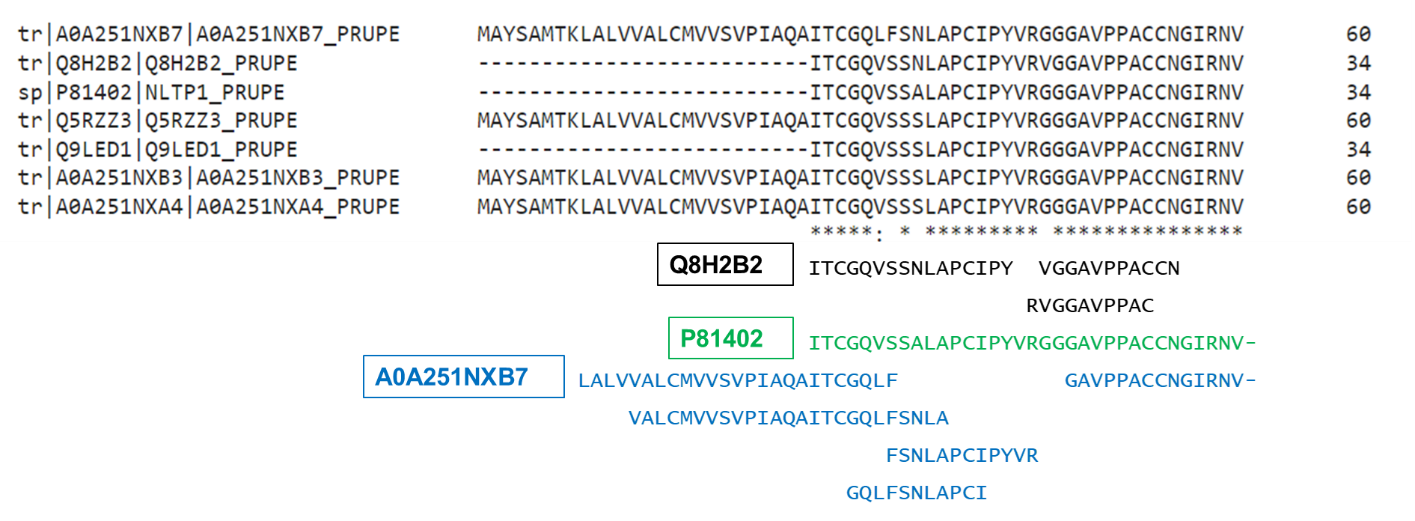

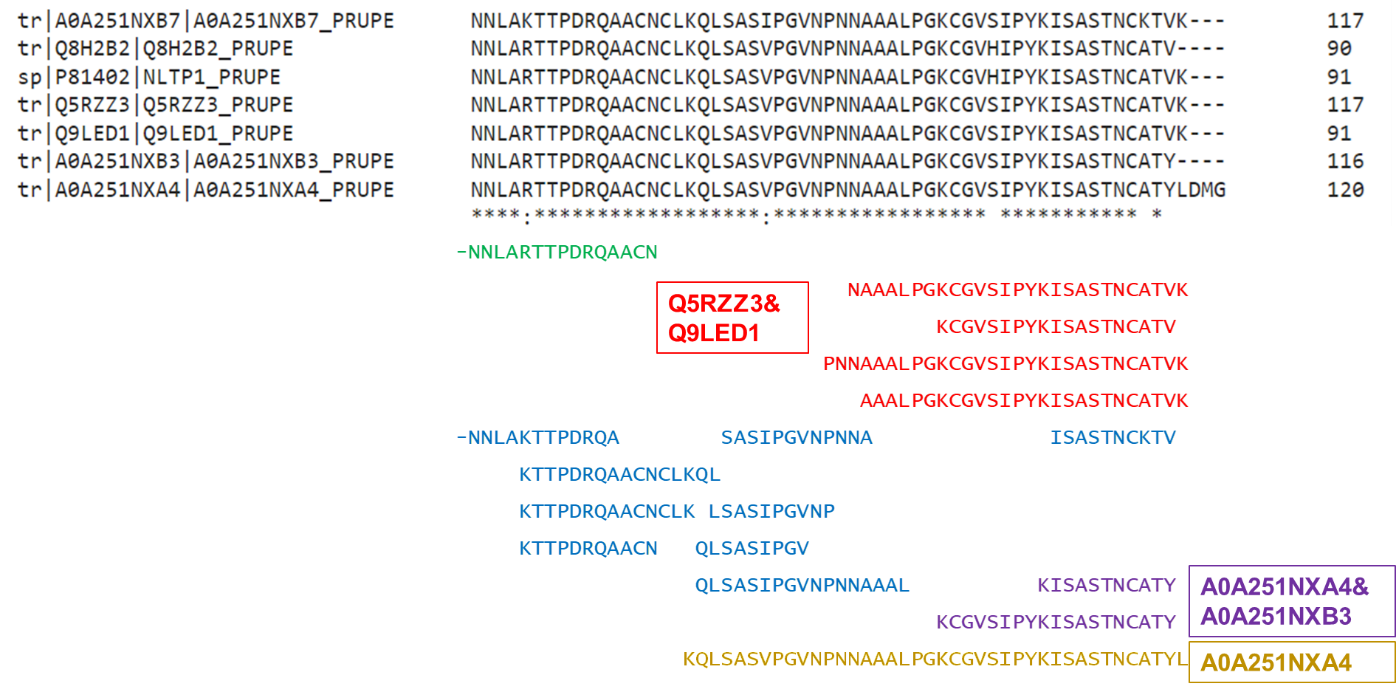
**

**
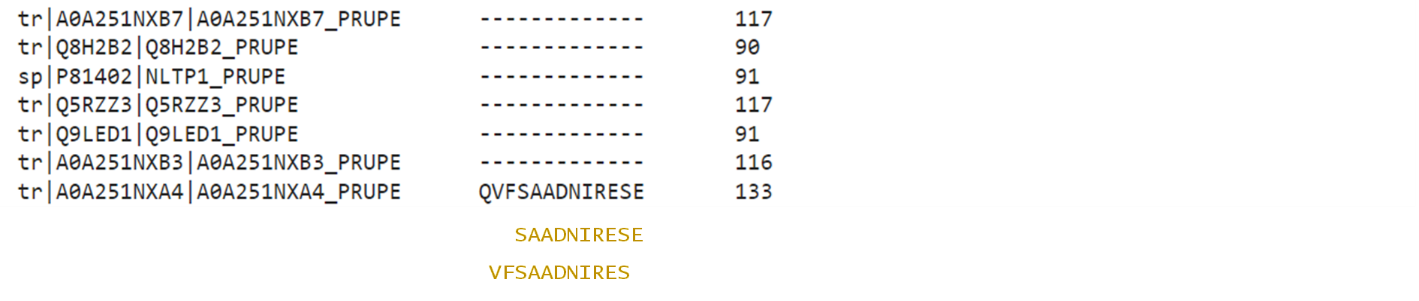
**

**Figure S7. Peptide sequence alignment of Pru p 3 isoforms and the unique peptides used to identify the isoforms from MS data (non-reduced).** Sequences were aligned using Clustal Omega and the unique peptides are indicated in gold for UniProt accession A0A251NXA4.

**
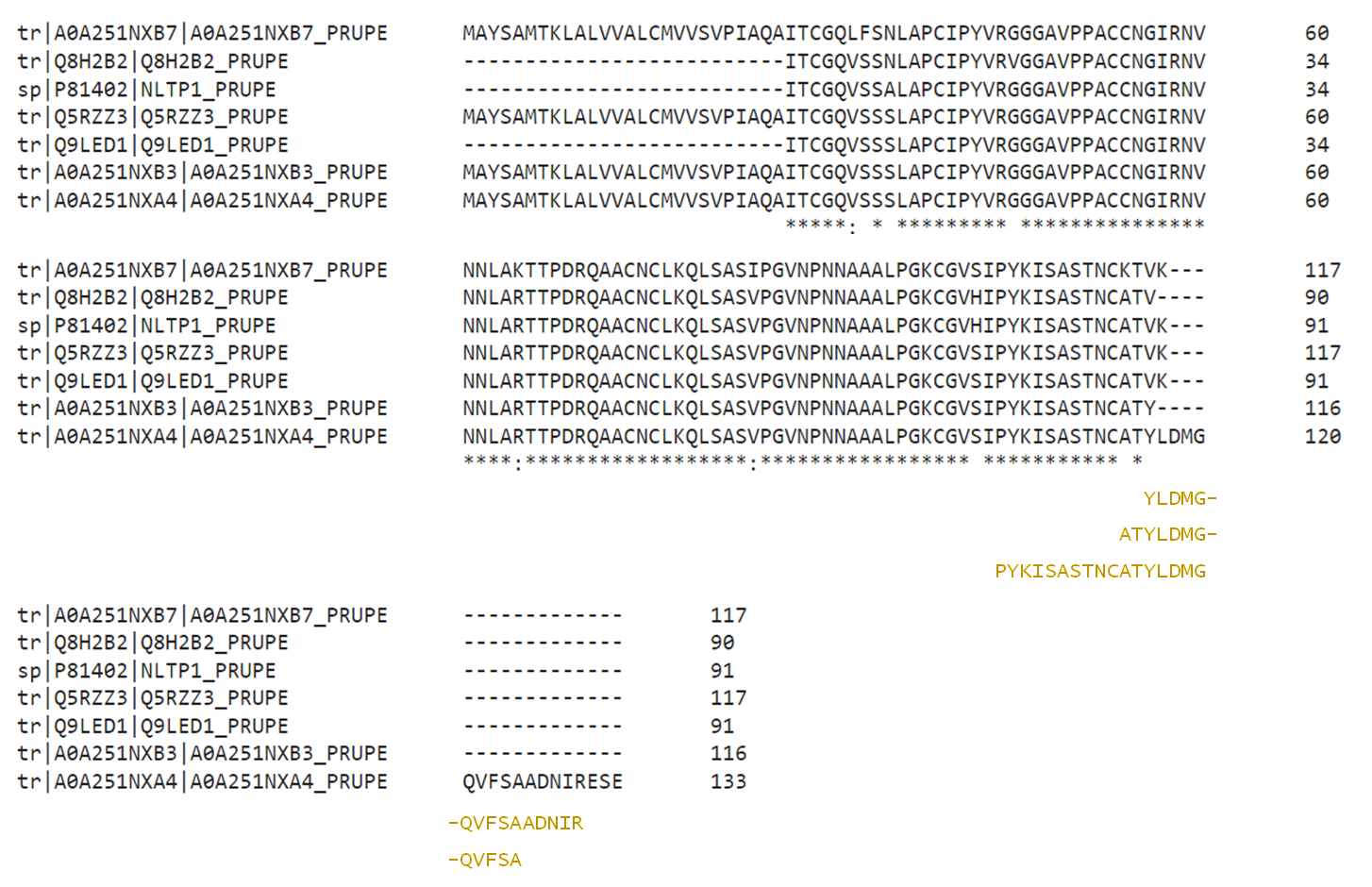
**

**Figure S8. Example extracted ion chromatograms for phosphorylated peptides in the reduced digests of Pru p 3.** Residue numbering is based on Uniprot accession Q9LED1 with phosphorylated serine residues underlined in bold.

^78^PYKI**S**AST^85^


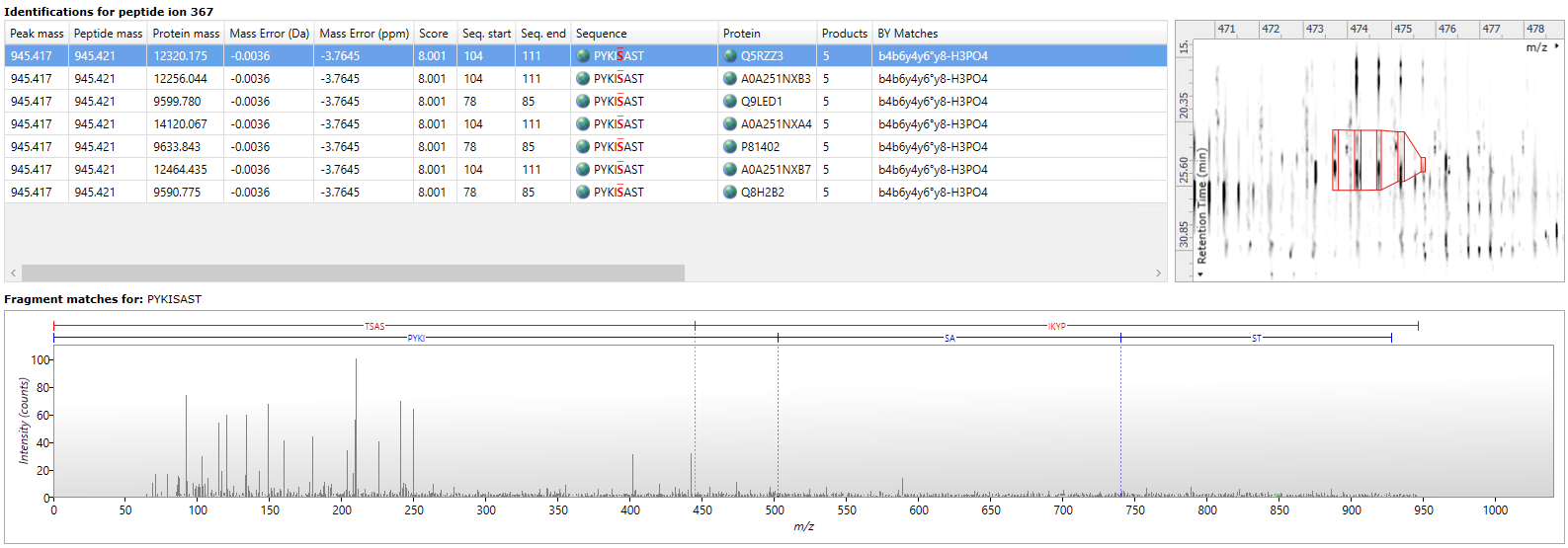


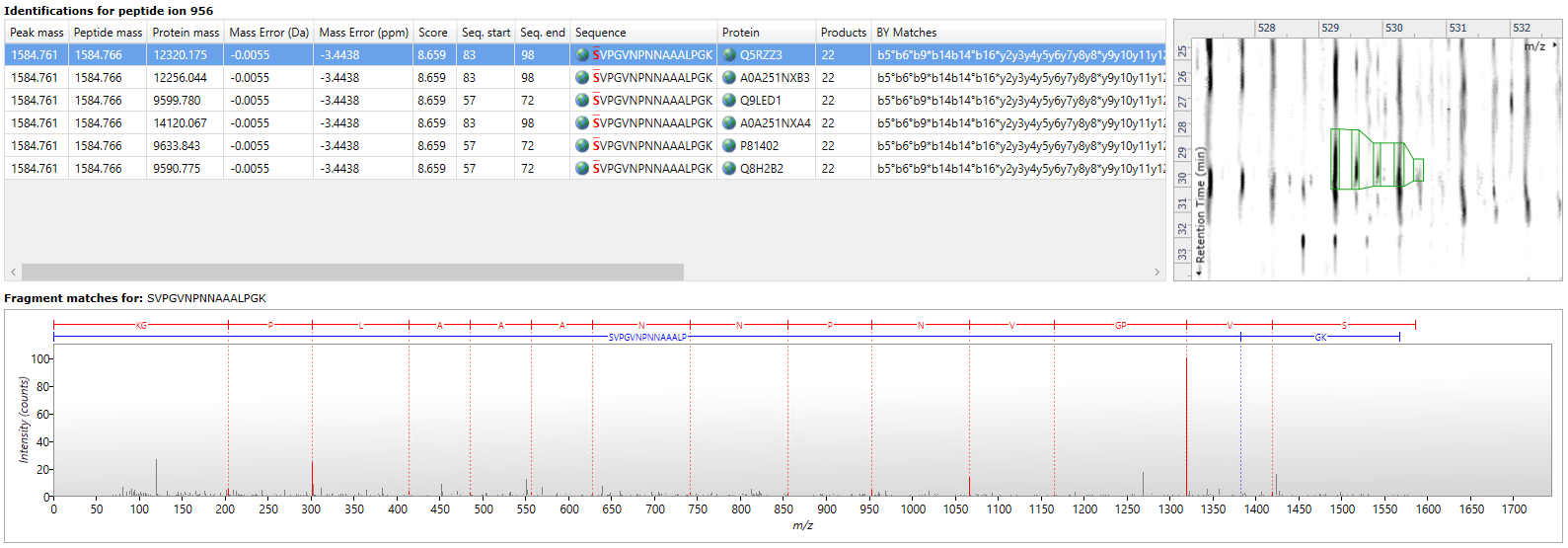
**^57^ S**VPGVNPNNAAALPGK **^72^**

^51^LKQL**S**ASVPGV^61^


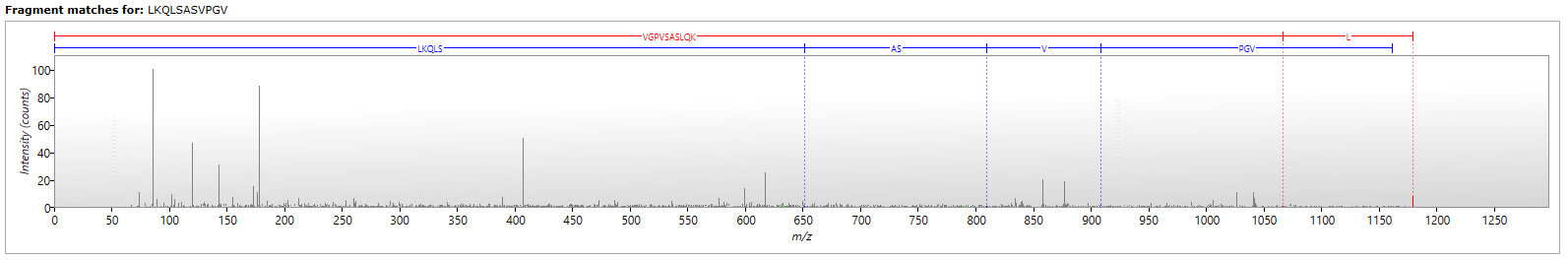


**Figure S9. IgE immunoblots of digests performed using sera from peach allergic patients**

A - Undigested Pru p 3; B - Pru p 3 digests after intestinal digestion at pH 8.0 high enzyme level with bile salts; C - Pru p 3 digests after intestinal digestion at pH 8.0 high enzyme level without bile salts. M: molecular maker. Lanes were as follows: lane 1 – reduced intact Pru p 3; lane 2 - non-reduced intact Pru p 3; lane 3 - reduced digests without bile salts; lane 4 - non-reduced digests without bile salts; lane 5 - reduced digests with bile salts; lane 5 – non-reduced digests with bile salts.


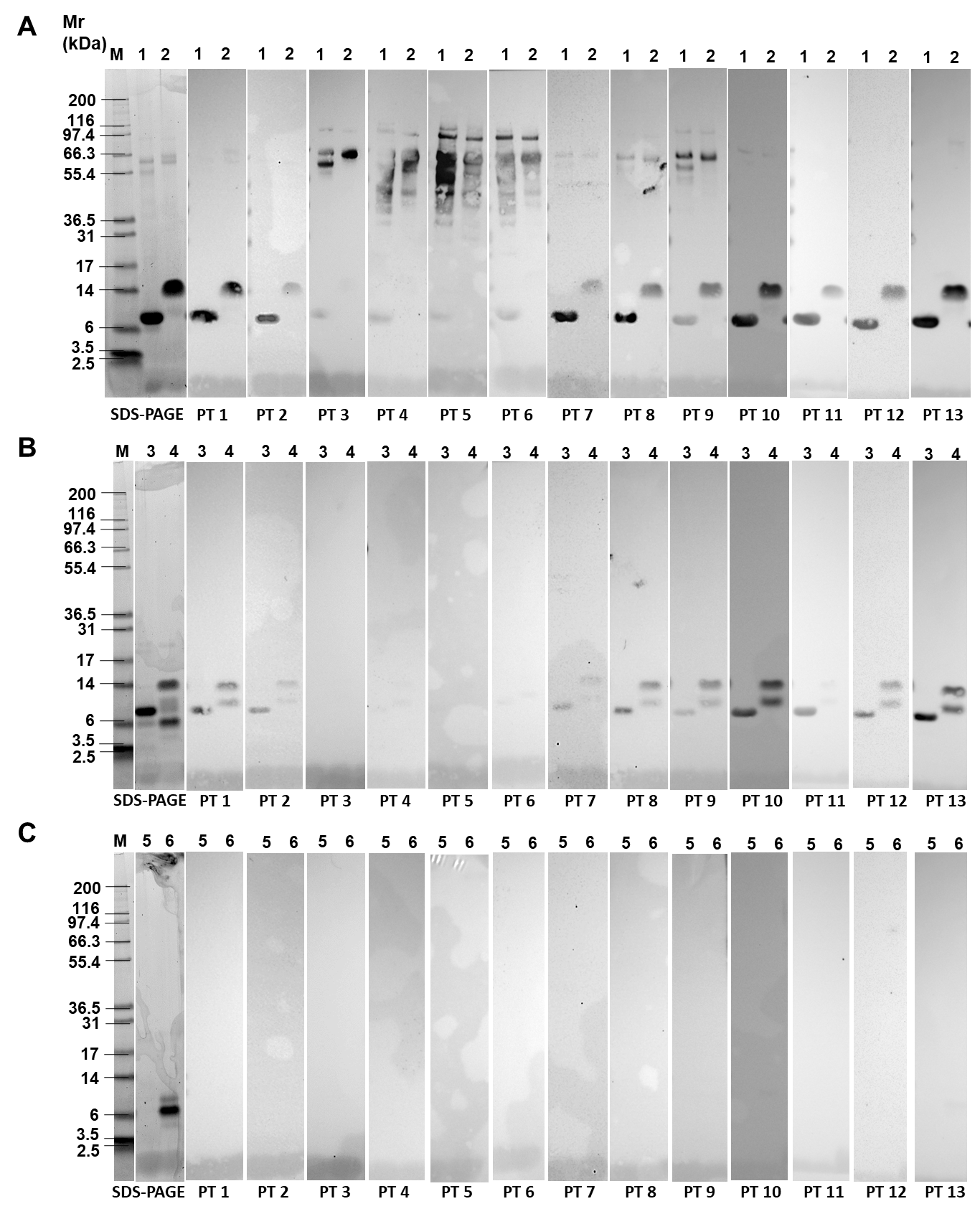


**Table S1.** **Summarised patient clinical information.** Symptom severity according to Sampson (add ref). Specific IgE was determined using either Phadia-ThermoFisher Immunocap or ISAC. ISU- ISAC standardised units; M- male; F- female. ISU - ISAC standardized units. OAS – oral allergy syndrome.

| **Patient No.** | **Gender** | **Age (Years)** | **Total IgE (**UI/mL**)** | **Pru p 3 specific IgE (kU/L)** | **ISAC sIgE Pru p 3 (ISU)** | **Symptoms** | **Symptom severity** |
| --- | --- | --- | --- | --- | --- | --- | --- |
| 1 | M | 24 | 3228 | 6 | 75.21 | Urticaria | 2 |
| 2 | F | 53 | 6,528.0 | 11 | 31.67 | OAS, urticaria | 2 |
| 3 | F | 33 | 640.1 | 6 | 24.57 | Anaphylaxis (urticaria+facial angioedema+dyspnea) | 4 |
| 4 | M | 25 | 195.8 | 6 | 11.88 | OAS, urticaria/facial angioedema | 2 |
| 5 | F | 36 | 440.8 | 7 | 10.45 | Urticaria | 2 |
| 6 | M | 27 | 85.8 | 4 | 9.93 | Urticaria | 2 |
| 7 | F | 51 | 308.7 | 8 | 6.12 | Anaphylaxis (urticaria+dyspnea) | 4 |
| 8 | M | 30 | 77.6 | 9 | 5.86 | Urticaria | 2 |
| 9 | M | 34 | 609.6 | 13 | 5.10 | OAS, urticaria/facial angioedema | 2 |
| 10 | F | 31 | 34.3 | 6 | 4.58 | Anaphylaxis (urticaria and dyspnea) | 4 |
| 11 | M | 22 | 304.5 | 6 | 4.16 | Anaphylaxis (urticaria, facial angioedema and dyspnea) | 4 |
| 12 | F | 44 | 64.9 | 6 | 2.96 | OAS | 1 |
| 13 | F | 26 | 166.4 | 0 | 2.56 | OAS | 1 |
